# Solving Navigational Uncertainty Using Grid Cells on Robots

**DOI:** 10.1371/journal.pcbi.1000995

**Published:** 2010-11-11

**Authors:** Michael J. Milford, Janet Wiles, Gordon F. Wyeth

**Affiliations:** 1 School of Engineering Systems, Queensland University of Technology, Brisbane, Australia; 2 School of Information Technology and Electrical Engineering and Queensland Brain Institute, The University of Queensland, Brisbane, Australia; New York University, United States of America

## Abstract

To successfully navigate their habitats, many mammals use a combination of two mechanisms, path integration and calibration using landmarks, which together enable them to estimate their location and orientation, or pose. In large natural environments, both these mechanisms are characterized by uncertainty: the path integration process is subject to the accumulation of error, while landmark calibration is limited by perceptual ambiguity. It remains unclear how animals form coherent spatial representations in the presence of such uncertainty. Navigation research using robots has determined that uncertainty can be effectively addressed by maintaining multiple probabilistic estimates of a robot's pose. Here we show how conjunctive grid cells in dorsocaudal medial entorhinal cortex (dMEC) may maintain multiple estimates of pose using a brain-based robot navigation system known as RatSLAM. Based both on rodent spatially-responsive cells and functional engineering principles, the cells at the core of the RatSLAM computational model have similar characteristics to rodent grid cells, which we demonstrate by replicating the seminal Moser experiments. We apply the RatSLAM model to a new experimental paradigm designed to examine the responses of a robot or animal in the presence of perceptual ambiguity. Our computational approach enables us to observe short-term population coding of multiple location hypotheses, a phenomenon which would not be easily observable in rodent recordings. We present behavioral and neural evidence demonstrating that the conjunctive grid cells maintain and propagate multiple estimates of pose, enabling the correct pose estimate to be resolved over time even without uniquely identifying cues. While recent research has focused on the grid-like firing characteristics, accuracy and representational capacity of grid cells, our results identify a possible critical and unique role for conjunctive grid cells in filtering sensory uncertainty. We anticipate our study to be a starting point for animal experiments that test navigation in perceptually ambiguous environments.

## Author Summary

Navigating robots face similar challenges to wild rodents in creating useable maps of their environments. Both must learn about their environments through experience, and in doing so face similar problems dealing with ambiguous and noisy information from their sensory inputs. Navigation research using robots has determined that uncertainty can be effectively addressed by maintaining multiple probabilistic estimates of a robot's pose. Neural recordings from navigating rats have revealed cells with grid-like spatial firing properties in the entorhinal cortex region of the rodent brain. Here we show how a robot equipped with conjunctive grid-cell-like cells can maintain multiple estimates of pose and solve a navigation task in an environment with no uniquely identifying cues. We propose that grid cells in the entorhinal cortex provide a similar ability for rodents. Robotics has learned much from biological systems. In a complementary way, in this study our understanding of neural systems is enhanced by insights from engineered solutions to a common problem faced by mobile robots and navigating animals.

## Introduction

Many animals demonstrate impressive navigation capabilities as they travel long distances in search for food and then unerringly return to their nests. Extensive experimentation has identified two primary mechanisms animals use to navigate – path integration [1], [2] and landmark calibration [3], [4]. Animals can update their estimate of location using self-motion cues such as vestibular input (path integration), and calibrate these estimates by sensing familiar landmarks such as visual cues (landmark calibration). Neural recordings from laboratory rats have revealed three types of spatially responsive neurons involved in path integration and landmark calibration: place cells [5], which respond to the rat's location; head-direction cells [6], [7], which respond to the rat's head orientation, and grid cells [8]–[11], which respond at regularly spaced locations in the environment. Outside of the laboratory however, in large natural environments, both these mechanisms are characterized by uncertainty: the path integration process is subject to the accumulation of error, while landmark calibration is limited by perceptual ambiguity. It is unknown how spatially selective cells respond in the presence of uncertainty when animals travel long distances.

In robotics, it has been well established that the uncertainty in measurements of self-motion and landmarks must be explicitly included when forming spatial representations of large real world environments [12], [13]. Probabilistic algorithms enable a robot to explicitly represent spatial uncertainty by simultaneously maintaining multiple estimates of a robot's conjunctive location and orientation (its pose) within its internal map. Each pose estimate can be updated by ideothetic sensory information, such as wheel encoder counts, until sufficient evidence from allothetic information gathered over time can strengthen one hypothesis over the others. One of the key advantages of being able to represent multiple estimates of pose is that even ambiguous sensory information becomes useful. While ambiguous cues will not immediately pinpoint the robot's exact pose, they can simultaneously maintain a subset of the possible current pose estimates, reducing the robot's uncertainly about its possible location. Given that wild rats effectively navigate in large and complex environments [14]–[16], it seems likely that they too have neural mechanisms that allow them to represent more than one estimate about their environment and spatial location.

In contrast to the pivotal place of uncertainty in robot navigation research, mechanisms for dealing with uncertainty have not been identified from cell recordings performed on laboratory rats. Three factors may be responsible: firstly, the small size and simplicity of the test enclosures may not engage or reveal such mechanisms; secondly, it is only possible to take simultaneous recordings from a limited number of cells out of more than 200,000 projection neurons in layer II of entorhinal cortex (of which only a fraction are actually grid cells) and 300,000 pyramidal cells in CA3 (place cells) [17], [18]; and thirdly, cell firing fields are averaged over extended periods of time, minimizing any evidence of transient encoding of multiple spatial hypotheses. Although the response of a specific cell can be characterized over the entire testing enclosure, it is not yet possible to determine with any certainty whether other unrecorded cells fired at the same time to represent some alternative estimate for pose. It remains unknown whether rats can maintain multiple estimates of their location and orientation in the environment.

In this paper, we present a brain-based robot model of navigation known as RatSLAM [19]–[21], which provides a novel insight into the functional significance of grid cells. Based both on rodent spatially-responsive cells and functional engineering principles, the cells at the core of the RatSLAM computational model have similar characteristics to rodent grid cells, which we demonstrate by replicating the seminal Moser experiments [8]. Based on our robot experiments in large real world environments, we hypothesize that *conjunctive* grid cells provide a computational mechanism for resolving measurement uncertainty in navigation by maintaining multiple estimates of location and orientation. We describe a new experimental paradigm designed to examine the behavioral and neural responses of a robot (or animal) in the presence of uncertainty. We apply the RatSLAM robot navigation model to this paradigm to show that conjunctive grid cells can encode multiple hypotheses of spatial location (and orientation) sufficient to localize a robot in a perceptually ambiguous environment, and analyze the neural activity underpinning the navigation performance. Finally, we discuss the implications of the research for future work in robotics and animal navigation research.

## Methods

### RatSLAM Model

**Figure 1:**
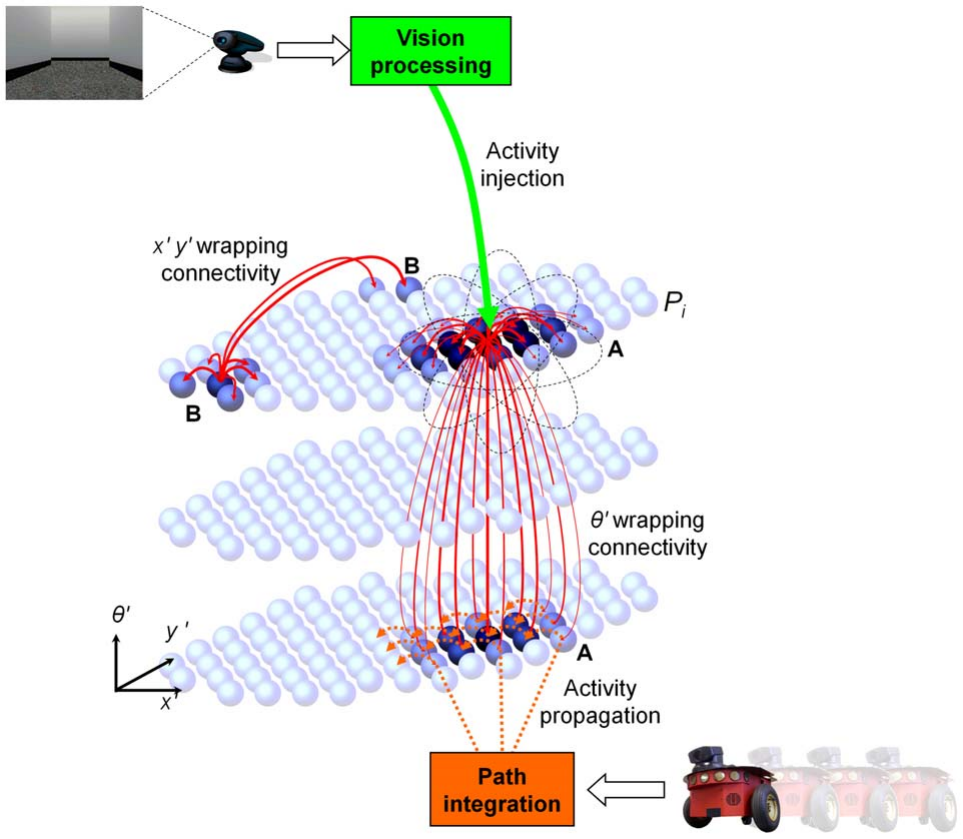
Schematic of the RatSLAM navigation model. The core RatSLAM navigation model consists of a continuous attractor network of location and orientation sensitive rate-coded cells. Each cell excites (solid line arrows) and inhibits (not shown) neighboring cells. A path integration module integrates robot movement information by shifting cell activity (dashed line arrows, only some shown for clarity reasons). When the robot sees a familiar visual cue, the vision processing system activates inputs into the cells associated with that visual cue (see Point A), enabling the robot to re-calibrate its estimate of its location in the environment. The layout of cells in the (x′, y′) plane starts off corresponding approximately to the (x, y) plane of the environment, but evolves under the influence of path integration and visual recalibration. The cell network can function in any tessellating layout (i.e. square, rectangle, hexagon), but is optimal in a hexagonal configuration (as shown), which minimizes the perimeter to area ratio and hence the number of wrapping connections for a given network size. In previous robot experiments the model has been shown to also function successfully with square or rectangular configurations [23].

We have developed a model of cells encoding spatial pose called RatSLAM that forms the key spatial representation for large-scale and long-term robotic navigation tasks [21], [22]. The RatSLAM model consists of a continuous attractor network (CAN) of rate-coded cells encoding the robot's location and orientation (analogous to grid cells in entorhinal cortex) and an episodic spatial representation (analogous to hippocampus) that is used to perform advanced navigation [23], [24]. For the work described in this paper, only the core continuous attractor network is relevant. At the heart of the model is a cluster of cells that forms a three dimensional continuous attractor network, with connections wrapped at the edges (Figure 1). Cells are connected to proximal cells by both excitatory and inhibitory connections, with connections on the edges wrapped around in a manner similar to that employed in the place cell model of McNaughton [2]. Within the context of sophisticated models that have been developed explicitly to model grid cells (which RatSLAM was not), RatSLAM is closer in characteristics to the attractor network models [25] rather than the interference models [26]. In the Discussion we provide a comparison of the RatSLAM model to the major continuous attractor models of grid and other spatial cells.

As well as being regulated by internal dynamics, the cell activity profile can change through a process of path integration when the robot is moving, and also when the robot sees familiar visual cues. The path integration process uses vestibular information from the robot's wheel encoders to shift activity in the network. Sensory input in the form of processed visual images is linked with co-activated cells through simple associative links. When these same visual cues are seen again, the linked cells are activated, and with enough visual input can reset the activity state of the entire cell network (Figure 1).

One complete iteration of the network consists of a full cycle of internal network dynamics, path integration and visual input. The number of cells is pre-determined, so their level of re-use grows with the size of the environment. New cells do not form; instead existing cells are recruited into the representation of the environment when the path integration process shifts activity into them.

### Attractor Network Dynamics

The intrinsic attractor dynamics are designed to maintain a single activity packet in the CAN. Local excitatory connections increase the activity of units that are close in (*x′*, *y′*, *θ′*) space to an active unit, generating the main cluster. Inhibitory connections suppress the activity of smaller clusters of activity. For each cell, local excitation and inhibition is achieved through a 3D Gaussian distribution of weighted connections, as shown by the solid arrows in Figure 1. The distribution, *ε*, is given by:
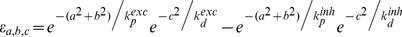
(1)where *k_p_* and *k_d_* are the variance constants for place and direction respectively, and *a*, *b* and *c* represent the distances between units in *x′*, *y′* and *θ′* co-ordinates respectively (constants are given in Table S1). The variances for inhibition are larger than for excitation, creating the so-called Mexican-hat function [27]. The connections wrap across all faces of the cell network, as shown by the longer solid arrows in Figure 1, so the indices *a*, *b* and *c* are given by:
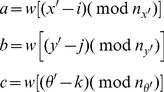
(2)where *w()* is a lookup function that provides the mapping between cells at opposite boundaries of the hexagonal plane. The change in a cell's activity level *ΔP* is given by:
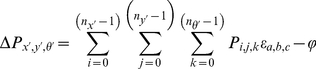
(3)where *n_x′_*, *n_y′_*, *n_θ′_* are the size of the network in number of cells along each of the dimensions *x′*, *y′*, and *θ′*, and the constant *ϕ* creates global inhibition. The final network stage thresholds activation levels in *P* to non-negative values and normalizes the total activation to one. When an experiment is started, a single cell unit is seeded with an activation level of 1, and then 5 network iterations are run in order to obtain a stable starting cluster of active cells.

### Path Integration

Although past versions of the RatSLAM model have used asymmetric weights to neurally perform the process of path integration, in this implementation path integration is achieved by displacing a copy of the current activity state by an amount based on nominal spatial areas and orientation bands of each cell. Copying and shifting activity offers stable path integration performance over a wider range of movement speeds and under irregular system iteration rates. Like the excitatory and inhibitory weight matrices, the path integration process can cause a cluster of activity in the cells to shift off one face of the cell structure and wrap around to the other. A nominal cell size dictates the rate at which activity is shifted under path integration, given in Table S1. For example, with a nominal cell size of 0.25 m×0.25 m, if the robot translates 0.25 meters, the network activity profile will shift by one unit in the (*x′*, *y′*) plane.

### Visual Processing of Landmarks

The path integration process is subject to the accumulation of errors in odometry, which becomes a critical problem over time. To correct path integration errors, RatSLAM learns unidirectional excitatory connections between its internal representation of visual landmarks seen at different bearings and ranges and cells that are active when those visual landmarks are seen. In this way, when a familiar visual landmark is seen, the learnt connections will activate the cells associated with seeing that visual cue, recalibrating the robot's internal estimate of its location and orientation. The connections between landmark representations and the cells are stored in a connection matrix *β*, where the connection between a particular visual cue *V_i_* and cell *P_x′_*_,*y′*,*θ′*_ is given by:
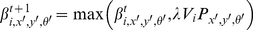
(4)where *λ* is the learning rate. When a familiar visual landmark is seen these connections are activated, resulting in a change in cell activity, *ΔP*, given by:
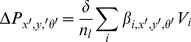
(5)where the *δ* constant determines the influence of visual cues on the robot's pose estimate, normalized by the number of visible landmarks *n_l_*. Figure 1 represents the moment in time when a familiar visual cue has just been seen, resulting in activation of cells associated with seeing that visual cue at a specific egocentric bearing and range, causing a shift in the location of the dominant activity packet (A). The previously dominant activity packet can also be seen (B).

### Robot Platform

**Figure 2:**
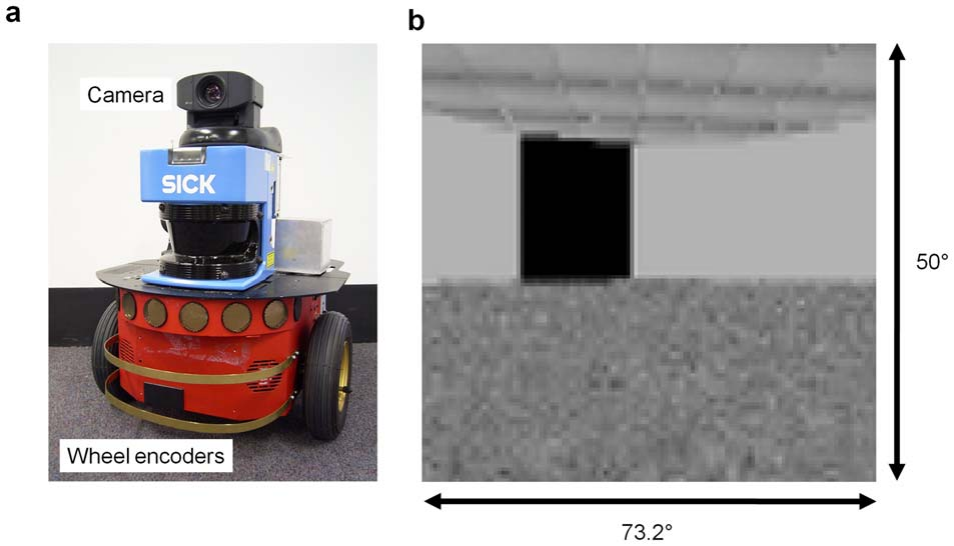
The simulated robot and sample visual field. (a) The real-life robot that was modeled was a Pioneer 2DXe from MobileRobots, with a forward facing camera providing the visual sensory input, and wheel encoders providing self-motion information. (b) A captured visual scene as the robot perceives the world in the circular arena, with approximately one cycle per degree visual acuity.

The virtual robot was modelled after a Pioneer 2DXe mobile robot from Mobile Robots Inc, with the relevant sensors being a forward facing camera and wheel encoders (Figure 2a). The robot's visual acuity was set at one cycle per degree to simulate that of a normally pigmented rat, although the field of view was less than that of a rat, at only 73.2 degrees horizontally, compared with approximately 270 degrees in the rodent (Figure 2b). The robot's sensor system was able to recognize rectangular uniformly colored visual cues and estimate their relative bearing and distance. The wheel encoders on the robot's wheels provided self-motion information in the form of translational and rotational velocities and formed the main source of vestibular information for the RatSLAM model.

### Replication of Grid Cells

Training and testing was performed in several virtual environments. All arena walls were 2.67 meters tall. All cues were rectangular flat cues attached to the exterior arena walls, measuring 0.5 meters in width and running the full height of the wall. In the two circular arenas (3.2 m and 1.6 m diameter) the robot was given random goal locations over a period of four hours to mimic a rat's collection of randomly thrown pieces of food. A single rectangular cue was attached at a fixed location to the arena walls. The sensitivity of the robot's path integration mechanism was varied to assess its impact on the simulated grid cell firing fields in the large circle. The attractor network had 2304 cells with 36 layers of 64 cells in each hexagonal layer. 10 network iterations per second were performed, so each four hour period consisted of 144000 network iterations.

#### Cue rotation experiments

The robot was trained for a period of four hours in the large circular arena with a fixed cue location. The cue was then rotated 90 degrees in a clockwise direction and the robot was allowed to explore the arena for a further four hours. The cue was then rotated back to its original position, followed by another four hours of exploration.

#### Darkness experiments

The robot was trained for a period of four hours in the small circular arena with a fixed cue location. All lighting was then removed from the arena, and the robot was allowed to explore the dark arena for a further four hours. Lighting was then restored, followed by another four hours of exploration. The cue remained in the same location for the duration of the experiment.

### Navigation under Ambiguity

**Figure 3:**
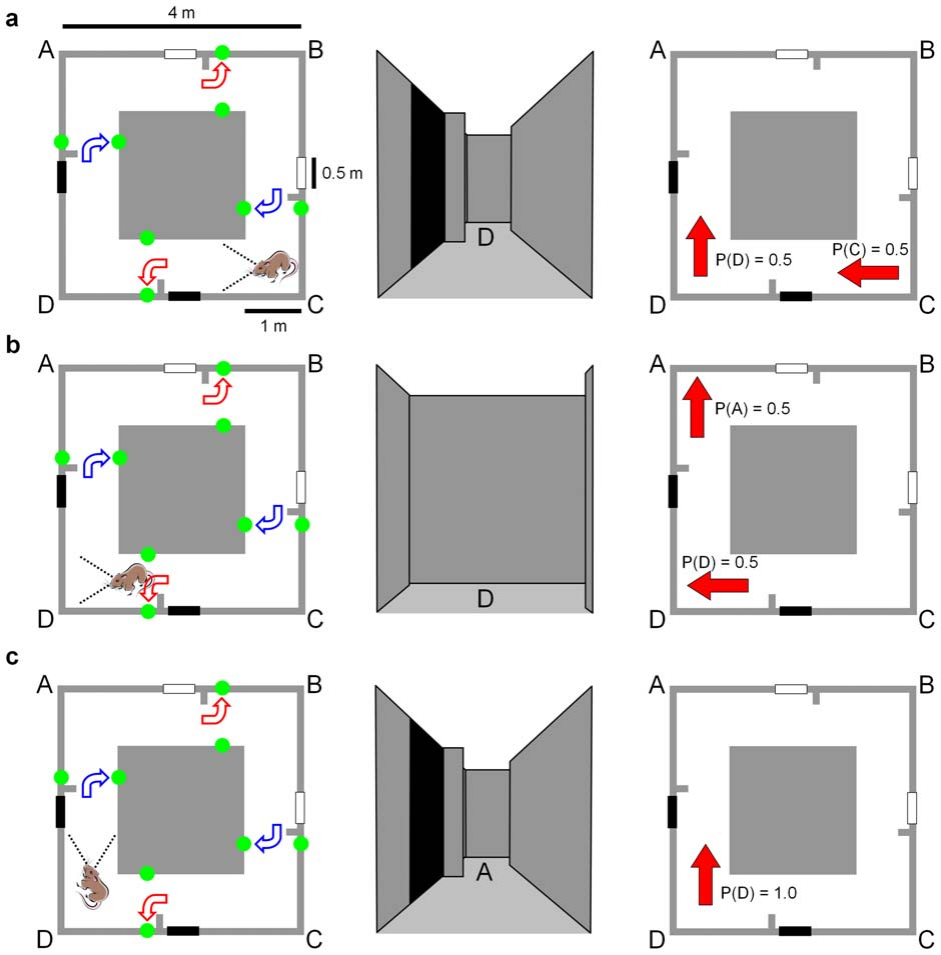
The perceptually ambiguous corridor arena and a schematic of the theoretically optimal navigation performance possible. A virtual rat is used to demonstrate the optimal navigation performance in the arena. The three columns show (in order from left to right) the plan view of the arena with rat location, the rat's view of the arena at each location, and the optimal probabilistic representation of the rat's location and orientation. Note the ‘blocker’ partitions that block the cues from sight once they have been passed (no such blockers were required for the robot due to its limited field of view). (a–b) Two equally weighted location hypotheses are maintained and updated until a second cue is sighted in (c), leading to a single correct location hypothesis. The curved arrows show the reward locations for the behavioral experiment.

We analyzed the navigational capabilities of the model in perceptually ambiguous situations using a square corridor arena measuring 4 meters in size with 1 meter wide corridors, with 2.67 meter tall interior and exterior walls (Figure 3a). The arena is designed such that every location has a twin in another part of the arena; the cues available at both locations are identical, meaning that perceptual sensors cannot distinguish between the two locations. The cues are rectangular flat cues attached to the exterior arena walls, measuring 0.5 meters in width and running the full height of the wall. Because the arena is small and simple in its layout, a robot can use dead reckoning to learn the arena's spatial layout and landmark locations. However, if that robot is removed and then replaced at a corner of the arena, it will have multiple hypotheses as to its location, and can only determine the correct location hypothesis by integrating sensory evidence over time as the robot moves. The attractor network was run at a higher rate of 14 iterations per second, in order to best capture the transitions between hypotheses.

We let a robot explore the virtual environment shown in Figure 3. During the exploration period, the robot was instructed that it would be rewarded at the four cue locations if it turned to look at the ‘correct’ wall for that location. The correct wall choice depended on location within the arena but not cue color – at one white cue the reward was dependent on the robot turning to look at the outer wall, while at the other white cue the reward was dependent on the robot turning to look at the inner wall (correct choices shown in Figure 3). Consequently cue color was not predictive of reward location.

After exploration, the robot was removed from the arena and then replaced in one of the corners. 10 trials were run for each of the four possible corner starting locations, for a total of 40 trials. The robot was not given any prior knowledge about its initial placement location. It was instructed to move around the arena in a clockwise direction and to try to obtain rewards at the cue locations. It was only given one chance at each cue location to be rewarded before being forced to continue moving. Each trial consisted of 4 decision trials, constituting one complete circuit of the arena. The typical duration of each exploration period and choice trial was 44 seconds.

### Driving Navigation Using Grid Cells

When the robot arrived at a cue location after training, it based its decision on which direction to turn to receive the reward on its internal estimate of its most likely location, in terms of which corridor it was most likely located in, as encoded by the ensemble cell firing. The most likely corridor, Z, was calculated by:
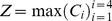
(6)where *C_i_* was the corridor occupancy likelihood for corridor *i*, given by:
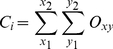
(7)where (*x_1_*, *y_1_*), (*x_2_*, *y_2_*) denote the corridor boundaries as indicated by the shaded areas in Figure S1, and the likelihood, *O*, of the robot being located in a particular discrete square spatial bin located at *(x, y)* is:
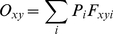
(8)where *P_i_* is the activity level of cell *i*, and *F_xyi_* is the discrete place field bin of cell *i* at location *(x, y)*. The bin size for the corridor arena experiments was 0.25 meters. The attractor network was scaled up to 10,368 cells with 36 layers of 288 cells in each hexagonal layer, with approximate cell numbers to environment size parity with the circular environments (circular environments – 2304 cells: 2m^2^ and 8m^2^ arena area, corridor arena – 10368 cells: 16m^2^ arena area).

## Results

### Cells Have Tessellating Firing Fields

**Figure 4:**
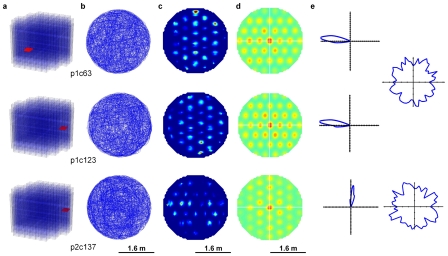
Cell firing field spacing and orientation was constant within the network. (a) The recorded cell locations. (b) The robot trajectories. (c) Cell firing fields (d) Firing field auto-cross correlations are shown. Firing field phase varied within the cell network, with only a few cells required to completely cover the environment. (e) Polar plots showing directional tuning of cells (left) and duration of time spent at each head direction (right). The first two rows show results for two cells from the same network, the third row shows a cell from a network with a different fundamental grid orientation.

To compare the spatial structure of firing fields in the model with firing fields in dMEC (Figure 1b in [8]), we tested the model in a circular enclosure with a diameter of 3.2 m. Cells developed a grid of regular firing fields (see Text S1 for field formation calculation) at locations corresponding to the vertices of a pattern of tessellating equilateral triangles spanning the environment surface (Figure 4c). Firing fields were clearly delineated from the background activity, but varied in strength between locations. Spatial autocorrelation analyses (see Text S1) of the activity distribution showed a regular tessellating pattern similar to that of grid cells [8], [10] (Figure 4d).

To examine the geometric characteristics of the grid, we repeated the analysis of the peaks in the autocorrelogram performed in the original study [8]. Within each firing grid, the distance from each peak to its nearest six peaks was almost constant (mean spacing ± s.d., s = 0.5±0.07 m). The angular separation between the vertices of the hexagon formed by each peak and its nearest six peaks was also in multiples of 60 degrees (mean separation ± s.d., 60°±13°). Field sizes were estimated by calculating the area of the peaks in the autocorrelogram exceeding a threshold of 0.2. Field sizes varied from 44 cm^2^ to 744 cm^2^ (mean area ± s.d., s = 267±159 cm^2^). Shrinking or expanding the environment had no effect on the spacing or size of the fields. The consistency in spacing, orientation and field size in a single network of cells matched the observed invariance in these properties at individual recording locations in dMEC [8]. All cells displayed strong directional tuning (Watson U^2^ test, U^2^ = 143.7 to 778.1, mean 586.7, see Text S1), with a mean angular standard deviation of 56.5° (Figure 4e).

To determine whether the model could replicate the observed increase in grid spacing and field size as distance increases from the postrhinal border, we recorded from three cell networks with varying sensitivity to motion cues (ideothetic cues). Field size and spacing varied jointly (correlation = 0.98), both increasing as the network sensitivity to robot motion decreased (Figure S2). Mean field size increased from 174 cm^2^ to 562 cm^2^, as field spacing increased from 34 cm to 73 cm. Gross variations in movement speed however had no effect on either field size or spacing.

### Grids are Anchored to External Cues

**Figure 5:**
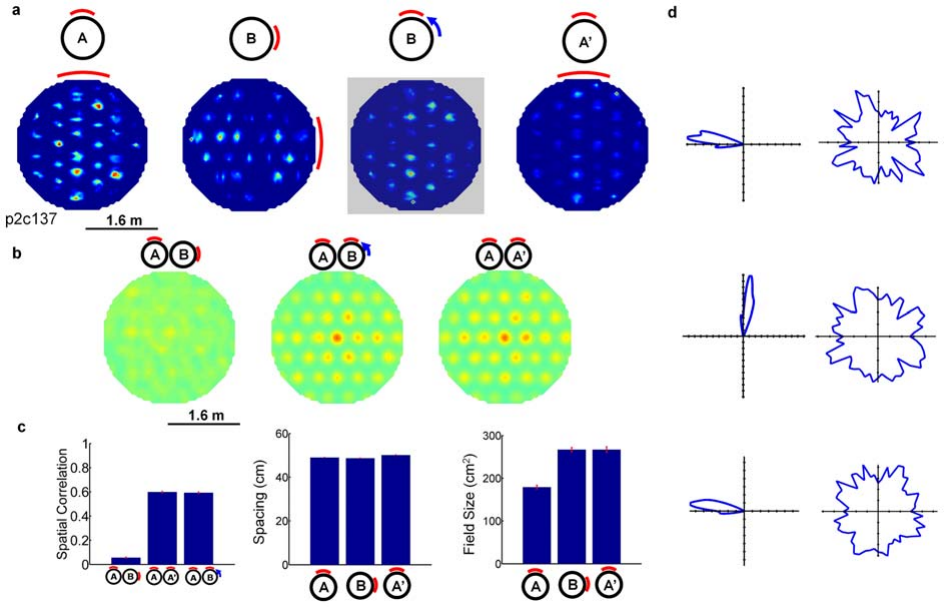
Firing fields under cue rotation, and in a second experiment with the original cue configuration. (a) Firing fields before and after rotation are seemingly unrelated, but become correlated if the B firing field is rotated 90 degrees counter-clockwise. (b) Cross-correlation plots corresponding to fields shown in (a). (c) Spatial correlation, field spacing and size for the different cue configurations (means ± s.e.m.). (d) The directional tuning of a specific cell rotated 90° after the cue rotation, and rotated back in a second experiment with the original cue configuration (left), and the duration of time spent at each head direction (right).

To compare the effect of allothetic and ideothetic cues, we conducted a number of trials in a circular arena with a single cue card on the wall and no distal cues (see *Grids are anchored to external cues* [8]). The cell firing grids were stable in successive trials in the same arena, supporting the strong influence of allothetic cues found in the rat trials. To further test the influence of allothetic cues, we allowed the model to develop stable firing fields in the environment, then shifted the cue by 90 degrees along the arena wall. The firing fields before and after cue rotation were dissimilar (correlation = 0.057, see Text S1), but became correlated if one field was rotated by 90 degrees (correlation = 0.60, Figure 5b). When the cue card was returned to the original configuration, the firing fields returned to their initial configuration (correlation = 0.60). The directional tuning of cells rotated with the cue (Figure 5d). Field spacing remained constant through the cue rotation (Figure 5c). Field size increased after the rotation then remained constant.

### Grids Degrade after Cue Removal

**Figure 6:**
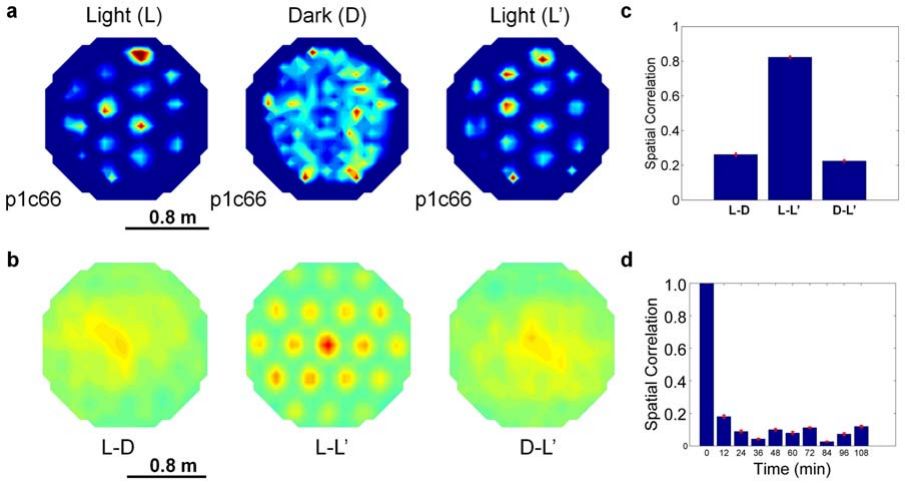
Firing fields drift in darkness and re-stabilize in the illuminated environment. (a) Firing fields in each experiment phase. (b) Cross-correlation plots of firing fields in light and darkness. (c) Spatial correlation of firing fields in light and dark periods. (d) Spatial correlation over time during darkness. Note the initial rapid decline in correlation. (all data, means ± s.e.m. See Text S1 for further details.)

In rodent experiments, grids were reported to be maintained in total darkness for 30 minutes (see *Grid structure persists after cue removal* [8]). In robot experiments however, allothetic cues are required to correct for path integration errors that accumulate over time. To test the effects of cumulative path integration error, we tested whether grids were maintained after all allothetic cues were removed. In sequential light-dark-light experiments, firing fields formed a regular tessellating pattern during the initial illuminated stage (Figure 6a). When the environment was darkened, firing fields steadily became irregular and distorted. The correlation between the new and original firing fields decreased as the time spent in darkness increased, dropping to a correlation of 0.1 within 24 minutes (Figure 6d). In the second illuminated stage, the original regular firing fields returned (correlation = 0.82). The period of darkness resulted in complete firing field degradation, even within the 30 minute duration of the original study. The disparity may be due to the role that non-visual cues play in maintenance of firing patterns in rodents (reported to be minimal [8]), or could be caused by path integration in the model being inferior to rodent path integration.

### Ambiguous Corridor Arena

**Figure 7:**
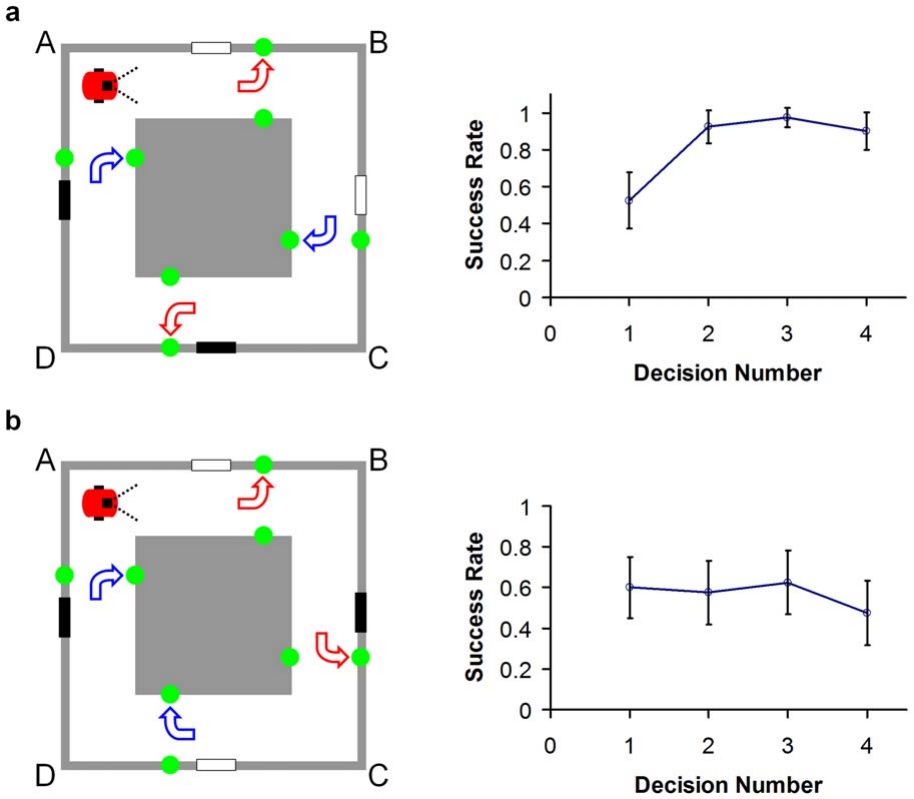
Robot behavioral performance in environments of varying perceptual ambiguity. (a) “Semi-ambiguous” corridor arena cue configuration. Reward directions specified during exploration and improving robot performance after replacement at choosing the correct reward direction. At the first decision point, by which time the robot has only seen one cue, performance is at chance. By the second decision point and beyond, however, the robot chose correctly approximately 90% of the time. (b) “Completely ambiguous” corridor cue configuration. Reward directions and steady robot performance with a theoretically indistinguishable cue configuration. Error bars show 95% confidence intervals. The robot performed no better than at chance.

In the behavioral task in the ambiguous corridor arena, when first replaced at a corner, the robot only had a 53% (n = 40 trials) success rate in obtaining a reward at the first cue it encountered (Figure 7a). However, by the second cue the success rate increased to 93%, and remained high for subsequent cues (98% and 90%). To help identify the effect of the visual cues on performance, we repeated the experiments in the same environment with a completely ambiguous cue configuration (Figure 7b). The success rates were not significantly different from chance (first to fourth cue encountered; 60%, 58%, 63%, 48% respectively), and did not improve as the robot encountered subsequent cues after the first.

**Figure 8:**
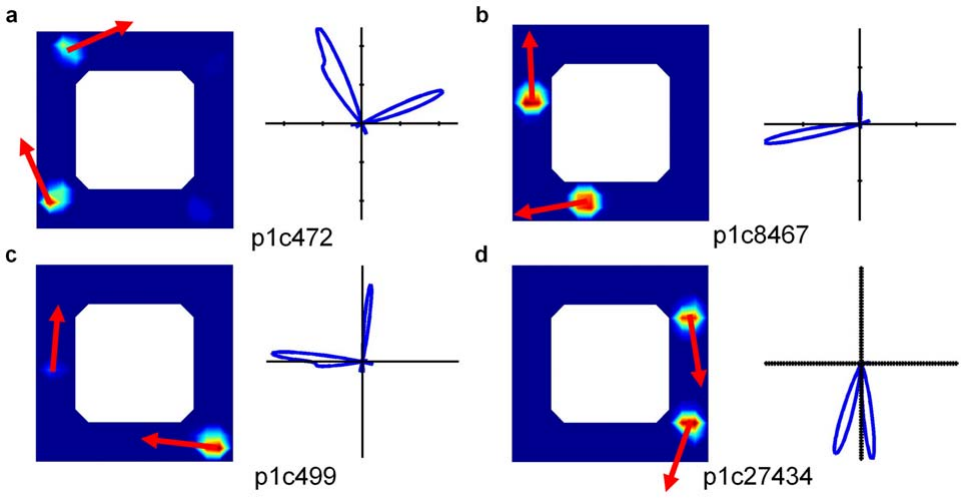
Cells encoded for both multiple distinct robot locations and robot orientations. Firing fields and directional tuning of four cells for the experiment shown in Figure 7a. The arrows superimposed on the location firing fields show the two robot orientations encoded by each cell, which correlate with the expected robot orientations at those locations during clockwise movement through the corridors. (a–c) show cells that each encode multiple locations and orientations in different corridors, while (d) shows a cell that encodes for multiple locations and slightly different orientations in the same corridor.

**Figure 9:**
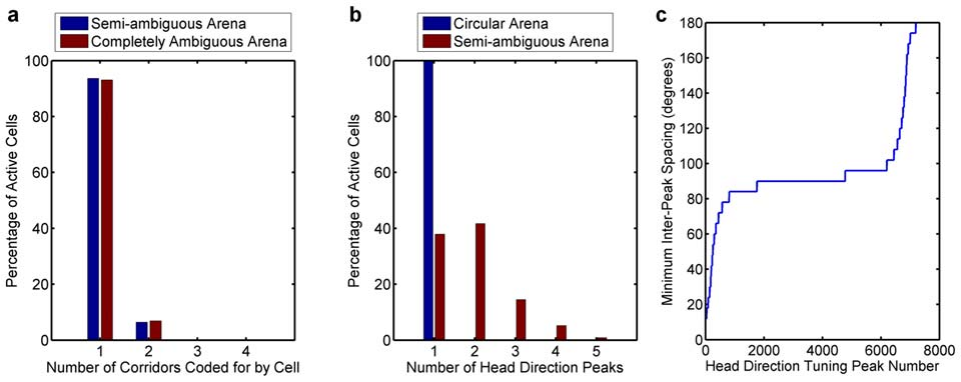
Place field distributions and tuning curve properties for the cell populations in the experimental arenas. (a) More than 6% of cells that were active at some time during the 80 trials encoded locations in two or more different corridors (semi-ambiguous: n = 83551 cells, completely ambiguous: n = 83460 cells). (b) Directional tuning of the cell populations was almost entirely unimodal in the circular arena experiment described in Figure 4 (n = 2304 cells). However, in the corridor arena, more than 60% of active cells encoded more than one distinct robot orientation (n = 5006 cells). (c) Distribution of the minimum inter-peak spacing for all cells encoding multiple robot orientations in the semi-ambiguous corridor arena (n = 3111 cells).

We next analyzed the firing fields and directional tuning of the cells (Figure 8). Although some cells displayed the characteristic grid-like firing pattern within one maze corridor (Figure 8b, p1c27434), other cells fired in multiple corridors (Figure 8b, p1c8467). Furthermore, many cells had bimodal directional tuning curves. To evaluate whether firing fields in different corridors was typical, we analyzed the population statistics over all 80 trials. The percentage of cells in the entire network that had firing fields for the semi-ambiguous and completely ambiguous corridor arena configurations were 20.2% and 20.1%, respectively. While the majority of these active cells coded for locations in only one corridor, more than 6% of active cells coded for locations in two different corridors, as shown in Figure 9a (calculated using Equation 7 with a threshold of *C_i_>0.01*).

To further analyze the orientation tuning curves and compare the level of uncertainty in the cell population's encoding of the robot's orientation in the circular open field environment and ambiguous corridor environment, we analyzed the *orientation* tuning curves of each cell (each cell in general encoded multiple locations as expected for grid-like cells) (Text S1). In the 1.6 meter circular arena, almost every cell that was active encoded only one distinct robot orientation (Figure 9b). In stark contrast, in the ambiguous corridor arena, more than 60% of active cells encoded more than one distinct robot orientation, with more than 20% encoding three or more distinct orientations.

As can be seen in p1c27434 in Figure 8b, cells could have multiple distinct peaks in their orientation tuning curves which encoded slightly different robot orientations in the same corridor. To confirm whether the multimodal tuning curves were primarily due to slightly separated orientation peaks in one corridor only, we constructed the distribution of minimum inter-peak angular distances for every cell with a multimodal tuning curve for one of the ambiguous corridor trials (Figure 9c). More than 75% of the inter-peak angular distances were between 80 and 100 degrees, confirming that most of the multimodal tuning curves encoded the robot's orientation in different corridors, rather than within the same corridor where robot orientation was highly similar.

**Figure 10:**
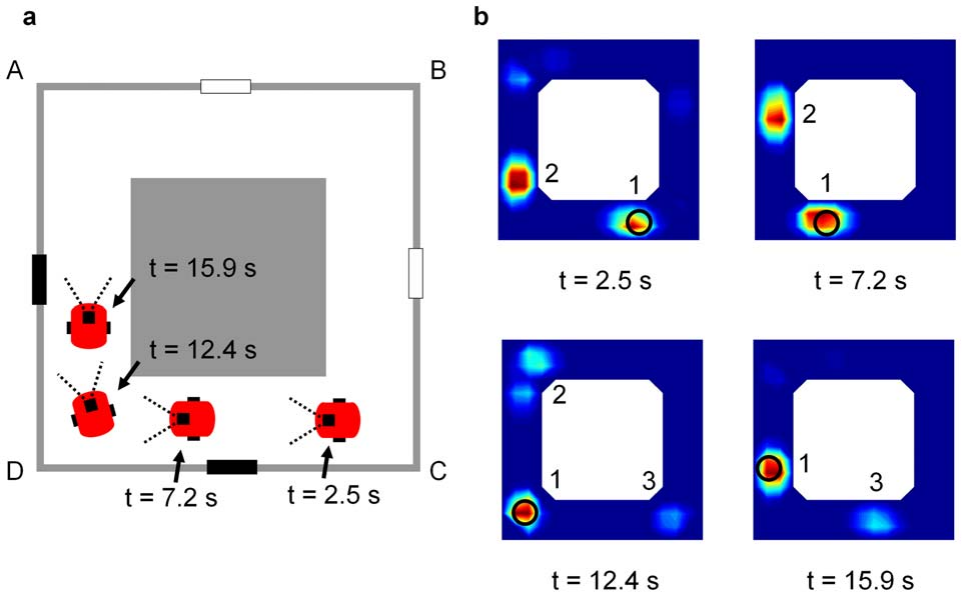
The actual robot pose and corresponding location estimates encoded by the ensemble cell firing. Each firing plot corresponds to various times after the robot was replaced at corner C facing D. (a) Schematic of the robot's pose corresponding to each of the four ensemble firing plots. (b) Location estimates as encoded by the weighted sum of the firing fields of all active cells at various times. The circle shows the robot's actual location. Cell firing initially (t = 2.5, 7.2 s) supported and maintained two approximately equal location estimates (1 and 2) – sighting the first black cue did not provide sufficient information to disambiguate the robot's location. After sighting of the second black cue (t = 12.4 s), cell firing resolved to code primarily for the correct location 1 – location estimate 2 disappeared (t = 15.9 s) and there was limited firing for a new location estimate 3. Figure 12 provides a schematic explanation of the ensemble cell firing.

By summing the place fields of all active cells weighted by cell activity, it was possible to visualize the robot's location estimates (Figure 10a) as encoded by the ensemble cell firing (Figure 10b, see Video S1). After replacement in the maze at corner C facing corner D (with a black cue visible), cell firing encoded approximately equally for corridors CD and AD (t = 2.5s, labeled ‘1’ and ‘2’ in the figure). As the robot moved along corridor CD, path integration shifted the location estimates (t = 7.2s) until the robot turned the corner at D (t = 12.4s). After sighting the second black cue, cells with firing fields encoding the correct location estimate 1 increased in activity, while cells with firing fields encoding the incorrect location estimate 2 reduced in activity. Cell firing also supported, but less strongly, a new location estimate 3 at C. After further robot movement (t = 15.9s), cell firing primarily supported the correct location estimate 1, to a lesser degree supported the new location estimate 3, and no longer supported location estimate 2.

## Discussion

Our results identify conjunctive grid cells as excellent candidates for the computational mechanism that addresses measurement uncertainty in spatial encoding. Our computational approach enabled us to observe transient population coding of multiple location hypotheses, a phenomenon which would not be easily observable in firing field plots from rodent recordings of a few dozen cells with data averaged over tens of minutes. Furthermore, the experimental arena and task provide a novel investigative tool for testing the neural and behavioral responses of navigating rodents in the presence of perceptual ambiguity.

For animal experiments with only behavioral analysis, but no neural recordings, we note that the experimental arena choice task shown in Figure 3 could be improved to make it more “schema-proof”. Currently, a schema of “see two of the same cues in sequence, turn inward, see two different cues in sequence, turn outward” would solve the task, while only localizing the animal to one of two possible locations. Having four reward locations at each cue, or changing the sequence of correct choices to (travelling clockwise in Figure 3 from the top white cue) white – turn outwards, white - turn inwards, black – turn inwards, black – turn outwards, requires any successful schema to uniquely identify the animal's location within the arena. Ideally, any rodent experiments would also involve neural recordings to more directly ascertain the rodent's spatial encoding during the experiment, and of course the robot in this paper was not given any ability to form schemas to solve the task.

### Comparison to Other Computational Models of Grid Cells

At the time of the invention of the RatSLAM system (2003), there was little biological evidence of any type of grid cell. While place cells were known to become directional over repeated traverses of long narrow corridors [28], there were no known cells with the inherent conjunctive properties of grid cells in layers III, V and VI, and no cells that were known to fire at regular spatial intervals. The robot model used conjunctive grid-like cells to handle perceptually ambiguous situations and to effectively use memory and computing resources through cell reuse [24], rather than to model a particular type of spatially selective neuron [23]. This difference in driving motivation – robot navigation in large and challenging environments, rather than high fidelity recreations of observed biological phenomena – is significant. However, it is still informative to compare and contrast RatSLAM with the other, primarily unembodied computational models of grid and other spatially-responsive cells.

The RatSLAM model falls under the continuous-attractor network class of grid cell computational models [2], [11], [25], [29], [30], as opposed to oscillatory interference models [26], [31]. The cells implemented in the RatSLAM model are rate-coded as opposed to spiking cells such as used in [32]. The number of neurons in the continuous attractor model (10368 in the corridor arena experiments, 2304 in the circular arena experiments) is comparable with other continuous attractor models such as [11] (16384 cells), but far greater than in independent neuron models such as [26]. While the core excitatory and inhibitory network dynamics are pre-wired rather than learned, associations between visual cues and internal spatial states are learned in an online manner.

In the model by [2], a default configuration of a square cell plane produces a rectangular grid of firing fields, rather than the triangular (hexagonal) grid found in rodents. To generate a triangular lattice grid firing field, a rectangular rather than square arrangement of cells can be used. In contrast, the RatSLAM model implements a hexagonal arrangement of cells with a cell count (288 in one plane) that enables symmetrical weight structures even with wrapping connectivity. To achieve irregular patterns like those possible in the Fuhs [25] model, the network connectivity would need to be structured differently. The cells in the RatSLAM model are also entirely conjunctive grid cells, rather than the place-only grid cells common in most other computational models. Consequently, relatively long training and testing times (4 hour segments in the circular arena experiment) can be needed in order to generate “complete” place field plots, as a cell will only fire if the robot is both located and orientated at the cell's preferred location and orientation.

The grid firing fields in RatSLAM rotate in response to cue rotation, like the model by [25] and unlike the model of [2]. The RatSLAM implementation described in this paper also only simultaneously implements one scale of grid. Where other theories postulate the combination of multiple grids with different periods to uniquely represent position, in our robot navigation experiments an additional episodic spatial representation is used to perform advanced navigation tasks [33]. In this paper we were able to simplify the task of navigation in the behavioral task by constructing from the ensemble cell firing an estimate of the most likely corridor the robot was located in, avoiding the need to combine multiple grids to construct a unique position estimate.

Unlike the conjunctive nature of the cells and their reuse through wrapping connectivity, without which the model cannot function successfully in large environments (both in these experiments and also robot navigation experiments [21], [22]), the network structure shape and hence geometry of the cell firing fields is not critical to the robot's navigation performance. This would suggest that the conjunctive and grid-like properties of the grid cells are functionally critical, while the hexagonal shape of the grid may be a computational optimality.

### Comparison to Place and Head-Direction Cells

Until the recent discovery of grid cells, the prime candidates for spatial representation in the rodent brain were place cells and head-direction cells. The techniques used for analyzing spatially responsive neurons – averaging cell firing over an entire experiment and recording from only a very small percentage of the cells – are not conducive to discovering whether place and head-direction cells temporarily fire to encode multiple locations and orientations. Using the arena, we can analyze whether it is even possible in principle given the neurophysiological separation of the two cell types.

**Figure 11:**
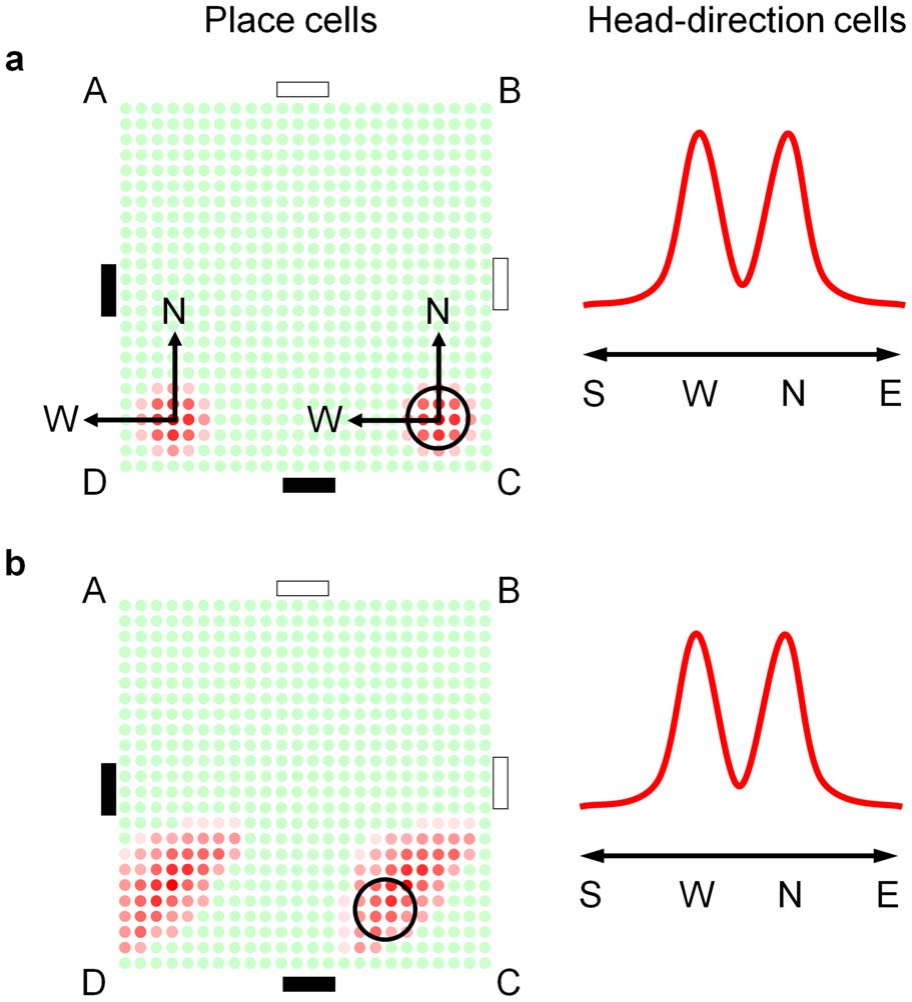
Hypothetical propagation of firing profiles of head-direction and place cells in the corridor arena. (a) The head-direction and place cell firing profiles that would be required to represent the multiple pose hypotheses for a rat placed at corner C facing towards corner D. The mesh of place cells is arranged to cover the entire experimental arena, with each place cell positioned in the location it encodes. The (N)orth and (W)est arrows show the two orientation estimates encoded by the head-direction cells, superimposed on the place cells. Without a conjunctive representation, there is no way to encode which orientation estimate is associated with each location estimate. (b) One possible propagation of place cell firing during movement from C to D without spatial memory binding between head-direction and place cells.

Consider first the cell firing profiles required to represent the rat's estimate of location after being replaced in the environment at corner C facing corner D (but not knowing this) and having seen the black cue, so that 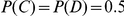 (Figure 3a). Two groups of place cells would need to fire, one group coding for location C and one group for location D. Two sets of head-direction cells would also need to fire to represent the two possible rat orientations separated by 90 degrees (Figure 11a). As the rat moved one corner clockwise, place cell firing would need to update to represent the movement of the two location estimates, one from C to D, and one from D to A. The update would require that the firing rates of place cells encoding location C gradually shift to cells encoding locations *west* of C (and hence closer to D). At the same time, the firing rates of cells encoding location D would need to gradually shift to cells encoding locations *north* of D (and hence closer to A). Mature place cells, however, generally have no directional attributes (with exceptions such as on linear tracks [34]), so the orientation information that dictates the direction that place cell firing should shift would need to come from elsewhere, such as the head-direction cells. Most importantly, the association would need to be specific so that the firing of cells encoding location C shifted *west* towards D rather than *north* towards B, and the firing of cells encoding location D shifted *north* towards A rather than *west* out of the arena (Figure 11b).

**Figure 12:**
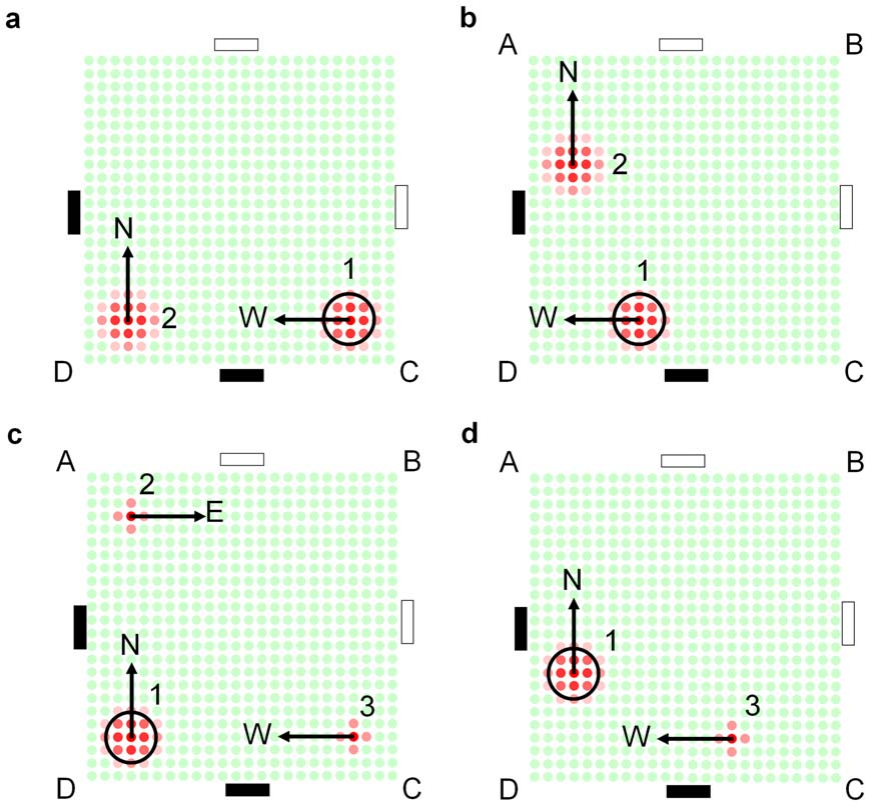
Schematic of the firing profiles of grid cells in the semi-ambiguous corridor arena experiment. The schematic mirrors the actual ensemble cell firing during the robot experiment (Figure 10). The robot's actual location is shown by a large circle. Because both orientation and location information is stored conjunctively with each grid cell, each cluster of active grid cells is associated with only one orientation estimate. (a–b) As the robot moves west, grid cell firing propagates to represent the movement of the two robot location estimates in two independent directions – one estimate moves west (1), the other moves north (2). (c) When the robot turns at corner D, only one existing location estimate (1) is supported by the sighting of a black cue, and a new location hypothesis (3) is created at C. Because the existing location hypothesis (1) has been further supported by sensory evidence, it is stronger than the newly formed location hypothesis (3). (d) The firing of cells encoding the unsupported location estimate (2) reduces and eventually ceases, leaving the dominant correct location estimate (1) and a secondary location estimate (3).

The neurophysiological separation of the place and head-direction cells [5], [7] renders this requirement a spatial memory form of the binding problem [35]. While mechanisms such as synchronization have been proposed as a way of binding the information of different neurons in other domains [36], the conjunctive properties of the recently discovered grid cells offer an elegant solution to the spatial binding problem, as demonstrated in this work. Figure 12 shows a schematic mirroring the movement of the location estimates encoded by the ensemble cell firing during the actual experiment. The bound place and orientation information stored in conjunctive grid cells enables independent and correct propagation of the location estimates encoded by the ensemble cell firing (Figure 12a–b). When the robot turns corner D (Figure 12c), it sees a second black cue. Seeing a black cue is consistent with only one of the two current location estimates (labeled ‘1’), and the firing of cells encoding the unsupported estimate (labeled ‘2’) reduces. Cell firing also supports a new location estimate at C (labeled ‘3’). The existing location estimate 2, which has been further supported by sighting of the second black cue, is more strongly supported by cell firing than the newly formed location estimate 3. After further robot movement (Figure 12d), the firing of cells encoding the unsupported location estimate 2 ceases, leaving the dominant correct location estimate 1 and a secondary location estimate 3.

### Role of Non-Conjunctive Cells

If conjunctive grid cells perform filtering of perceptual ambiguity, which is the role suggested in this paper, why then are only some grid cells in the rodent directionally tuned? In the ambiguous corridor arena experiments described in this paper, only some cells encoded locations in multiple corridors and multiple distinct robot orientations, while others encoded only a single orientation and place. Many navigational situations do not require a rat to maintain and update multiple estimates of its pose – often there are unique visual, tactile, olfactory, or auditory cues which immediately disambiguate the rat's location. Some environments are small enough that the rat's vestibular system provides sufficiently accurate path integration information over the short-term to navigate ambiguous sections successfully. Furthermore, while a location may be adequately represented with only a few non-directional grid cells and a few head-direction cells, many more directional grid cells are required to represent every possible orientation of the rat at that location. Conjunctive cells may uniquely provide the rat with the computational mechanism required to navigate in ambiguous environments, but perform a more integrated role in simpler environments with many distinct cues. The axonal projections from layers III and V in EC, where the majority of conjunctive cells are located, to the non-directional grid cells in layer II, may provide a location-only read out of the multiple pose estimates stored in the conjunctive cells [10].

Based on our experience in creating robot navigation systems, and the current state of recording technology, we envisage future research on perceptual ambiguity in navigation to combine robotics and neuroscience. Firstly, experience from robot experiments in real world environments can be used to guide the design of new experimental paradigms for animals. In this paper, we have presented a new paradigm inspired by robot experiments that can only be solved if a robot (or rat) can at least temporarily maintain and correctly update multiple estimates of their location. Secondly, if rodents do indeed encode multiple coherent location and orientation estimates for short periods of time, current place field (and head direction cell preference) reconstruction techniques would not show them due to the averaging of firing rates over many minutes. One key advantage of a computational neural model is the ability to reconstruct neural population codes at any time during an experiment, using every neural unit, rather than a small selection. Embodied robot models such as RatSLAM provide a means by which to explore the functional implications of neural firing, such as navigation performance, that cannot be provided by recording techniques alone.

### Conclusion

Extensive robot navigation experimentation in real world environments has shown that being able to represent uncertainty is a necessity for effective navigation. In the robotics domain this ability is provided by probabilistic algorithms that facilitate the maintenance of multiple estimates of robot pose. We have shown that a model of conjunctive grid cells in an autonomous robot performs an analogous role, allowing it to navigate in a perceptually ambiguous environment. Conjunctive grid cells may similarly provide rats with a solution to the fundamental navigation problem of dealing with uncertainty, allowing them to navigate effectively in the real world environments they encounter every day.

## Supporting Information

Figure S1 Schematic of the place field binning and corridor zones used in the occupancy likelihood equation. The lightly shaded rectangles show the areas used to calculate the corridor occupancy likelihood, and the dashed grid shows the place field bins, each 0.25 meters square.
(0.69 MB TIF)

Figure S2 The sensitivity of a cell network's path integration process to motion cues affects field size and spacing. (a) Movement trajectories. (b) Firing fields. (c) Average field sizes (d) Field spacing. The lower the sensitivity to ideothetic sensory information, the larger the resultant field sizes and spacing (all data, means ± s.e.m.).
(2.91 MB TIF)

Table S1 RatSLAM parameter values. The RatSLAM continuous attractor network and visual learning system use a number of parameters that ensure stable network dynamics.
(0.03 MB DOC)

Text S1 Cell recording and processing. This text describes the techniques used for cell field formation, the field size and spacing calculations, the error bar calculations, the spatial autocorrelation and crosscorrelation calculations, and the directional tuning analysis.
(0.08 MB DOC)

Video S1 Video of the multiple pose estimates encoded by the ensemble cell firing (see Figure 10). After initial placement at corner C (bottom right) facing corner D (bottom left), the robot sees a black cue and cell firing encodes two equally likely location estimates. These location estimates are updated as the robot moves towards corner D. After turning corner D and seeing a second black cue, the cell firing supporting the correct location estimate strengthens, while the cell firing supporting the incorrect estimate weakens and then ceases.
(3.64 MB AVI)
