# Supplementary figures and images for: Solving Navigational Uncertainty Using Grid Cells on Robots
Source: PLoS Comput Biol. 2010 Nov 11;6(11):e1000995. doi: 10.1371/journal.pcbi.1000995 (PMC2978698; doi:10.1371/journal.pcbi.1000995)

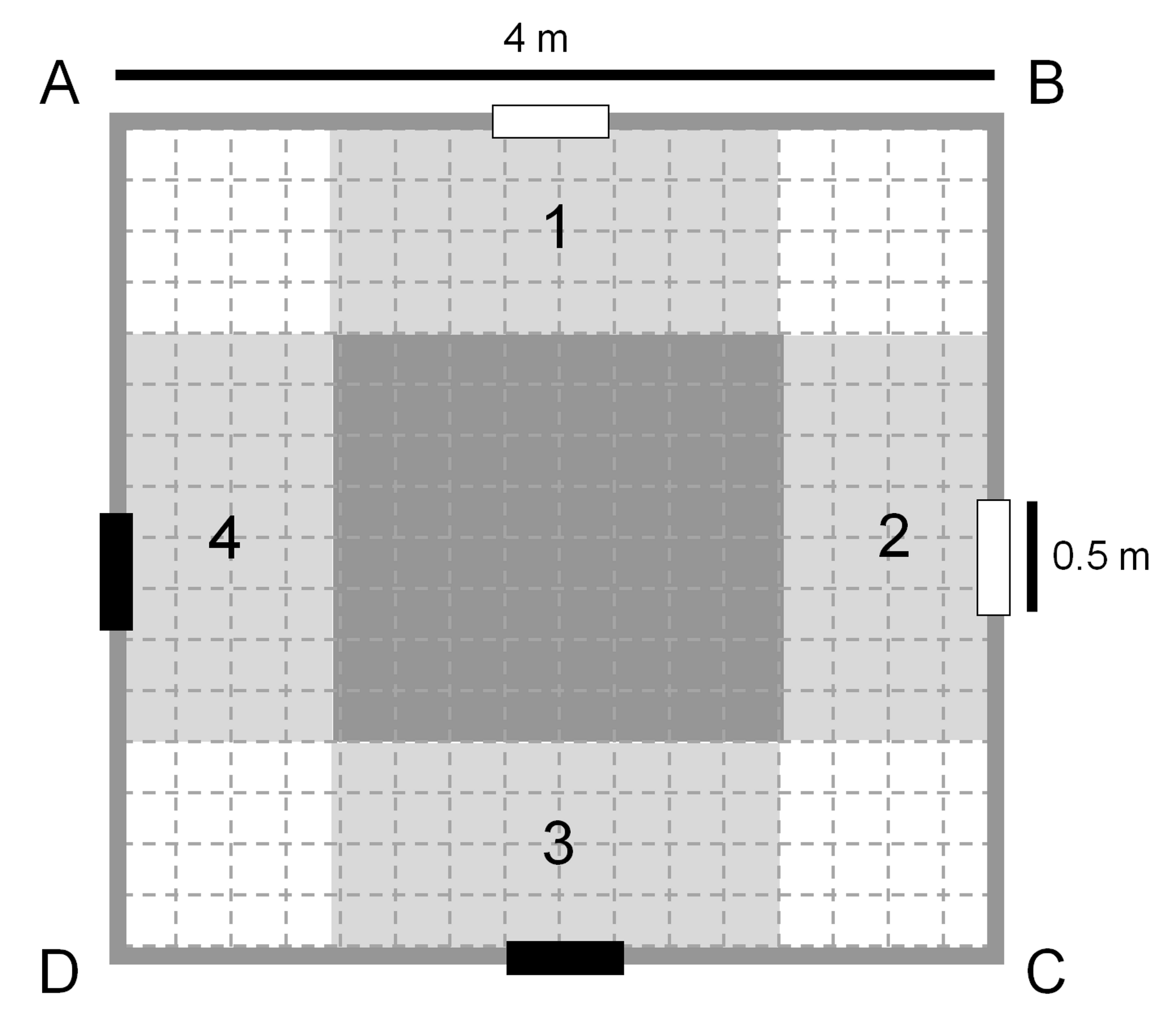

Supplement: Figure S1 — Schematic of the place field binning and corridor zones used in the occupancy likelihood equation. The lightly shaded rectangles show the areas used to calculate the corridor occupancy likelihood, and the dashed grid shows the place field bins, each 0.25 meters square. (0.69 MB TIF) [file pcbi.1000995.s001.tif]

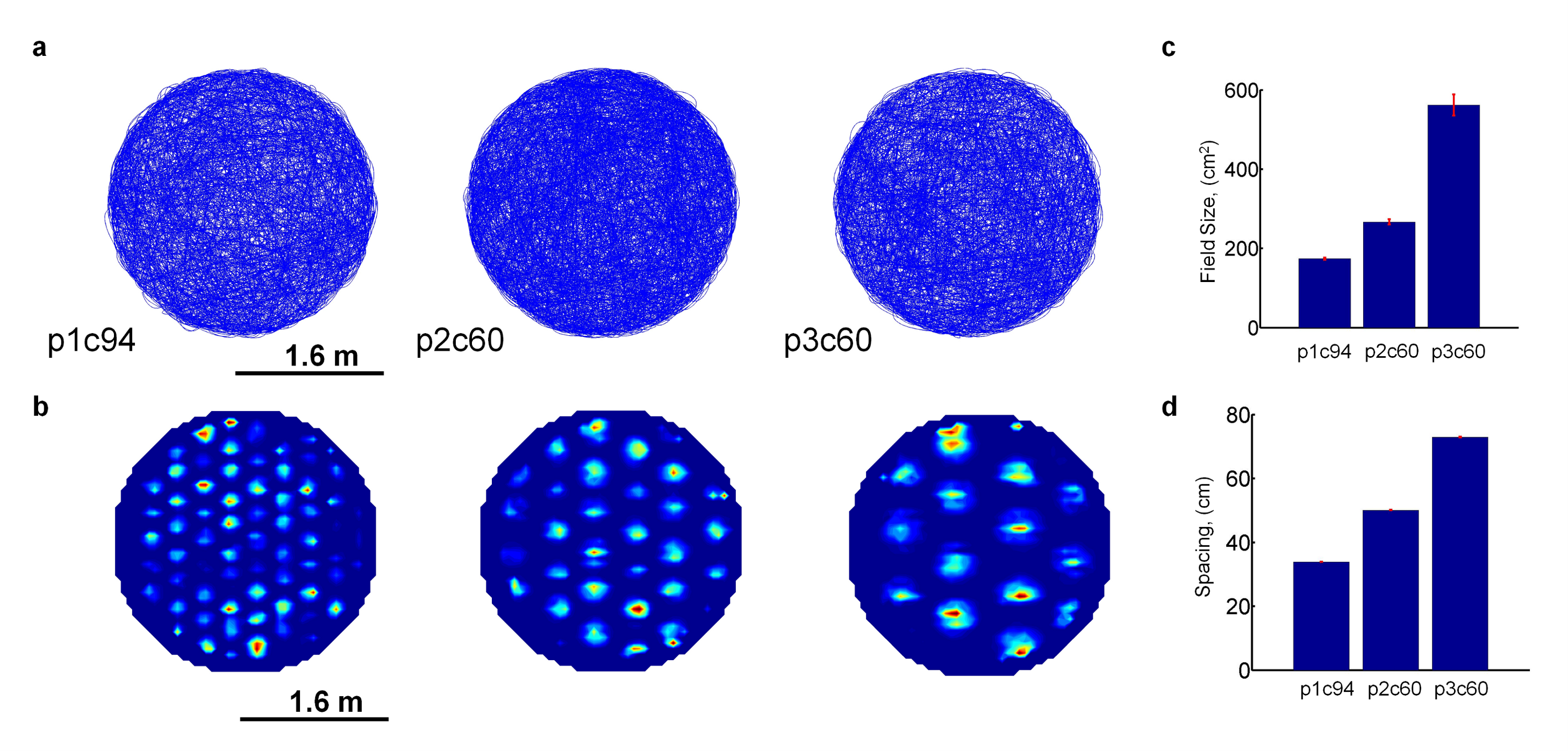

Supplement: Figure S2 — The sensitivity of a cell network's path integration process to motion cues affects field size and spacing. (a) Movement trajectories. (b) Firing fields. (c) Average field sizes (d) Field spacing. The lower the sensitivity to ideothetic sensory information, the larger the resultant field sizes and spacing (all data, means ± s.e.m.). (2.91 MB TIF) [file pcbi.1000995.s002.tif]
