# Supplementary material for: Solving Navigational Uncertainty Using Grid Cells on Robots
Source: PLoS Comput Biol. 2010 Nov 11;6(11):e1000995. doi: 10.1371/journal.pcbi.1000995 (PMC2978698; doi:10.1371/journal.pcbi.1000995)
Supplement: Table S1 — RatSLAM parameter values. The RatSLAM continuous attractor network and visual learning system use a number of parameters that ensure stable network dynamics. (0.03 MB DOC) [file pcbi.1000995.s003.doc]

| **Parameter** | **Value** |
| --- | --- |
| *k_p_*, *k_d_* (excitation) | 4 cells (1.0 m, 40°) |
| *k_p_*, *k_d_* (inhibition) | 8 cells (2.0 m, 80°) |
| Nominal cell size | 0.25 m × 0.25 m × 10° |
| *n_x'y'_*, *n_θ'_ (Circular arenas)* | 64, 36 |
| *n_x'y'_*, *n_θ'_ (Corridor arena)* | 288, 36 |
| *Φ* | 0.00002 |
| $\lambda$ | 0.36 |
| *δ* | 0.1 |
