# Supplementary material for: Solving Navigational Uncertainty Using Grid Cells on Robots
Source: PLoS Comput Biol. 2010 Nov 11;6(11):e1000995. doi: 10.1371/journal.pcbi.1000995 (PMC2978698; doi:10.1371/journal.pcbi.1000995)
Supplement: Text S1 — Cell recording and processing. This text describes the techniques used for cell field formation, the field size and spacing calculations, the error bar calculations, the spatial autocorrelation and crosscorrelation calculations, and the directional tuning analysis. (0.08 MB DOC) [file pcbi.1000995.s004.doc]

Solving Navigational Uncertainty using Grid Cells on Robots

Supplementary Information

Michael J. Milford^ab^ (corresponding author), Janet Wiles^b^ and Gordon F. Wyeth^ab^

^a^School of Engineering Systems, Queensland University of Technology, Brisbane 4000 Australia

^b^School of Information Technology and Electrical Engineering and Queensland Brain Institute, The University of Queensland, St Lucia, Brisbane 4072 Australia.

Cell recording and processing – circular arenas

The firing activity of all cells in the network was recorded during every experiment. As the cell model abstracts spike firing rates as continuous ‘activation’ levels, no spike sorting or clustering was required. The robot’s location was also recorded. Much of the analysis here is identical or similar to that performed in the original grid cell studies [1].

Field formation

The place fields of each cell were formed by normalizing the cumulative firing activity of each cell at each robot location in the environment by the total time spent by the robot at each location. Continuous robot occupancy information was converted into a discrete representation by sampling a square grid over the testing environments (resolution 6.6 cm in the circular arena, 25 cm in the corridor arena). The mean cell activity level *λ* at location *x* (coordinates abstracted to a one-dimensional vector) was calculated as:

$\lambda_{x}=\sum_{i=1}^{n} f\left(x,r_{i}\right)\alpha_{i}/\max\left(\sum_{i=1}^{n} f\left(x,r_{i}\right),1\right)$ (1)

where *n* was the number of recordings per cell, *α_i_* was the cell activation level at time *t_i_*, *r_i_* was the location of the robot at time *t_i_*, and *f( )* is a linear smoothing function:

$f(a,b)=\left\{\begin{matrix} \left(1-\left|a-b\right|\right)/s, \left|a-b\right|<s \\ 0, \left|a-b\right|\geq s \end{matrix}\right.$ (2)

where *s* was the resolution of the occupancy grid.

Field size and spacing calculations

To determine the firing field size and spacing for a cell, the cell’s entire firing field map was first cropped to a central square measuring 32% of the total arena area, in order to avoid boundary effects. Firing peaks were extracted by searching for local maxima. Spacing data was obtained by measuring the distances to the six closest neighbouring peaks for each peak, over all cells. Angular variation data was obtained by measuring the bearing differences between adjacent peaks, relative to the centre peak. Field size data was extracted by searching contiguous areas around each firing peak where the firing rate was above 20% of the maximum firing rate [1].

Statistical Analysis

Error bars on graphs show the standard error of the mean. In most experiments all cells were recorded, resulting in large *n* values and small associated SEM values, especially when compared with biological data. For example, the *n* value for the *A* field size in Figure 5c is 45100, with an average field size of 174 cm^2^ and a standard deviation of 100 cm^2^, resulting in a SEM of 2.805 cm^2^.

Spatial autocorrelations and crosscorrelations

To determine whether the firing fields of the simulated grid cells formed a regular pattern, we calculated the spatial autocorrelation for each cell’s firing field. Crosscorrelations were used to compare the firing fields of different cells. The correlation coefficient *γ(u,v)* for any two cells was calculated as:

(3)

where $\lambda_{1}\left(x,y\right)$ and $\lambda_{2}\left(x,y\right)$ were the mean activation levels of cell 1 and 2 at location $(x,y)$*,* $u$ and $v$ were the spatial offsets, and $\bar{\lambda}_{1}$ and $\bar{\lambda}_{2}$ were the mean activation levels of cell 1 and cell 2 across all locations $(x,y)$. To calculate the autocorrelation for one cell, $\lambda_{2}\left(x-u,y-v\right)$ was replaced with $\lambda_{1}\left(x-u,y-v\right)$.

Directional tuning

Analysis of the directional tuning of cells was performed as in the study by Sargolini et al. [2]. In brief, the directional tuning graphs were obtained by plotting cell activity as a function of robot orientation divided into 60 bins of 6 degrees. The angular standard deviation was calculated by dividing the resultant vector across all 60 bins by the sum of the individual vector lengths. Watson’s U^2^-test was used to compare the distribution of robot orientations at the times when a cell was active with the distribution of robot orientations over the entire experiment.

Cell recording and processing – corridor arena

Detection of Distinct Peaks in Cell Orientation Tuning Curves

To calculate the angular spacing between peaks in multimodal orientation tuning curves, first any of the 6-degree-wide robot orientation bins *H_i_* with values below 0.001 of the total bin sum were discarded:

$\mathbf{H}=\max(\mathbf{H}-0.001\sum_{i} H_{i}, 0)$ (4)

Bins were then clustered into continuous segments, wrapping around 360 degrees. The number of clusters was counted, and for cells with more than one peak, the minimum inter-peak distance *d* was calculated:

$d_{ij}=\min\left( \left|\delta_{i}-\delta_{j}\right|, 360-\left|\delta_{i}-\delta_{j}\right| \right), i\neq j$ (5)

where *δ_i_* and *δ_j_* were the orientations associated with each peak pair.

References

1. Hafting T, Fyhn M, Molden S, Moser M-B, Moser EI (2005) Microstructure of a spatial map in the entorhinal cortex. Nature 11: 801-806.

2. Sargolini F, Fyhn M, Hafting T, Mcnaughton BL, Witter M, et al. (2006) Conjunctive Representation of Position, Direction, and Velocity in Entorhinal Cortex. Science 312: 758-762.
